# P-926. A Comparative Analysis of Infection-Related Mortality and Relapse Rate: Restarting vs. Continuing Antibiotic Regimens in Endocarditis Patients Post-Cardiac Intervention

**DOI:** 10.1093/ofid/ofae631.1117

**Published:** 2025-01-29

**Authors:** Ali Al Safi, Courtney Nichols, Courtney Hebert, Jianing Ma, Jing Peng, Mohammad Mahdee Sobhanie

**Affiliations:** The Ohio State University, Columbus, Ohio; OSU Wexner Medical Center, Columbus, Ohio; The Ohio State University, Columbus, Ohio; Ohio State University, Columbus, Ohio; The Ohio State University Wexner Medical Center, Columbus, Ohio; The Ohio State University, Columbus, Ohio

## Abstract

**Background:**

The duration of antibiotic therapy post cardiac intervention (PCI) for treatment of infective endocarditis (IE) remains controversial despite current clinical guidelines. There is paucity of data on the PCI antibiotic duration relating to pathogens, presence of an infected cardiovascular implantable electronic device (CIED), vegetation size, and duration of bacteremia. Due to these limitations, antibiotic therapy may be extended although the impact in clinical practice remains unknown.

**Figure:**
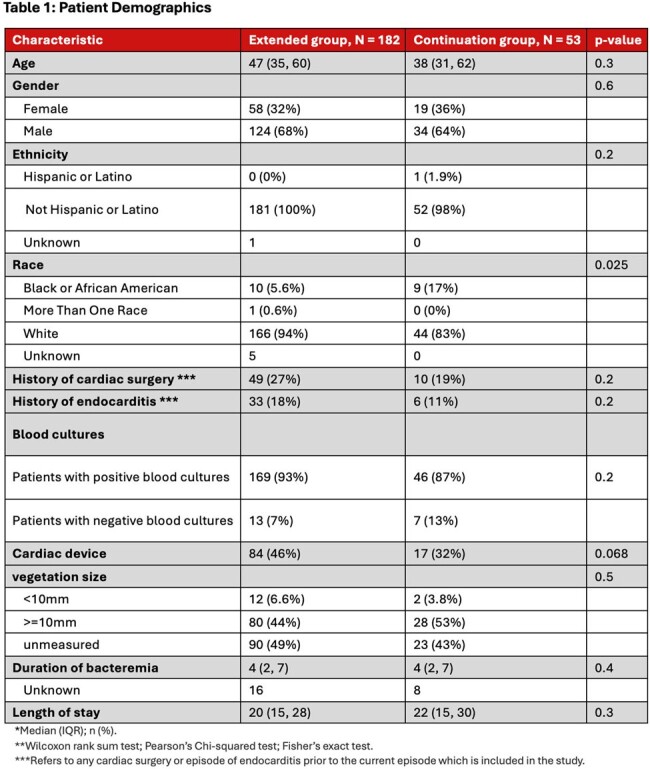

**Methods:**

This was a retrospective study comparing outcomes in patients with IE admitted to The Ohio State Wexner Medical Center who underwent valve replacement and/or CIED extraction from January 2013 to November 2017 based on whether the course of antibiotic therapy was restarted PCI (extended group) or if antibiotics prior to cardiac intervention were included in the total antibiotic duration (continuation group). Patients who completed the full course of antibiotics prior to cardiac intervention were excluded. The primary outcome was a composite of one-year infection-related mortality and IE relapse PCI. The proportional win-fractions regression model was used to compare the composite outcome between the groups.

**Figure:**
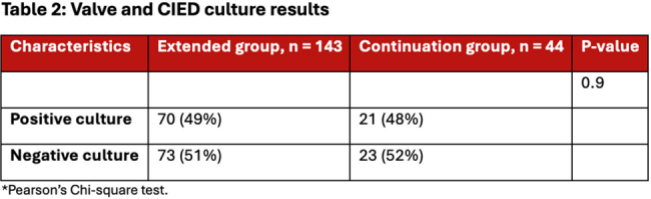

**Results:**

235 patients met study inclusion criteria, 182 in the extended group and 53 in the continuation group. Patient demographics are shown in **table 1**. 91.5% of patients had positive blood cultures (n = 215) with a median bacteremia duration of 4 days with the most common pathogen being *Staphylococcus aureus* as shown in **figure 1**. 79.6% of patients had OR cultures sent (n = 187) shown in **table 2**. The average antibiotic duration PCI was 39.7 days. 5 patients had bacteremia at the time of surgery. The study revealed no statistically significant difference between the two groups in the primary outcome (Win Ratio 1.59, CI: 0.80-3.15, p-value 0.18) shown in **table 3**.

**Figure:**
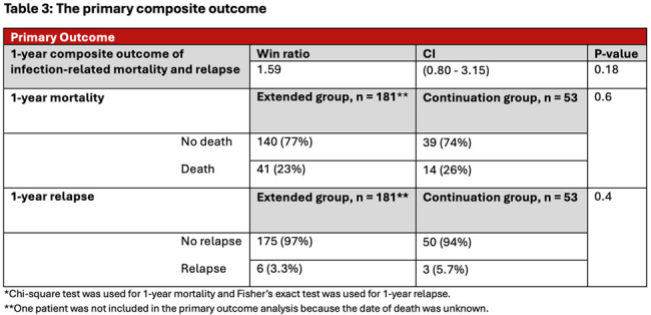

**Conclusion:**

For patients with IE requiring surgical intervention, the outcome of one year mortality and infection relapse was not significantly different between the two groups after adjusting for potential confounders. This study suggests that extending antibiotics may not be associated with improved survival or infection relapse, however prospective studies are needed to confirm these findings.

**Figure:**
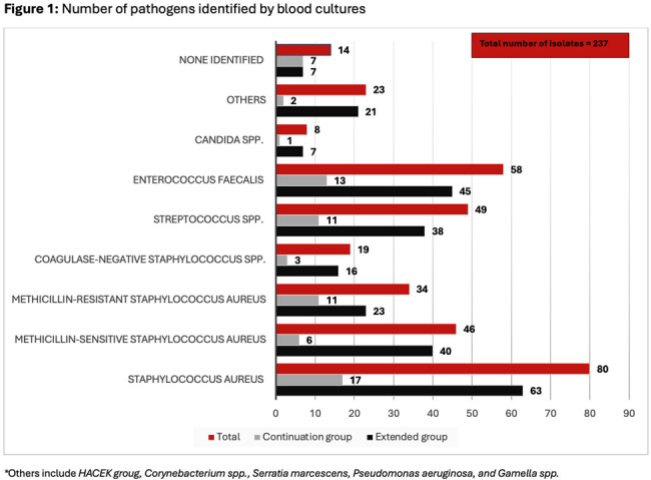

**Disclosures:**

**All Authors**: No reported disclosures

